# Genetic polymorphisms of *PCSK2* are associated with glucose homeostasis and progression to type 2 diabetes in a Chinese population

**DOI:** 10.1038/srep14380

**Published:** 2015-11-26

**Authors:** Tien-Jyun Chang, Yen-Feng Chiu, Wayne H-H. Sheu, Kuang-Chung Shih, Chii-Min Hwu, Thomas Quertermous, Yuh-Shan Jou, Shan-Shan Kuo, Yi-Cheng Chang, Lee-Ming Chuang

**Affiliations:** 1 Department of Internal Medicine, National Taiwan University Hospital, Taipei 10002, Taiwan; 2 Department of Bioinformatics and Biostatistics, National Health Research Institutes, Zhunan Town, Miaoli County 35053, Taiwan; 3 Department of Internal Medicine, Taichung Veterans General Hospital, Taichung 40705, Taiwan; 4 Division of Endocrinology and Metabolism, Taipei Veterans General Hospital, Taipei 11217, Taiwan; 5 Section of General Medicine, Department of Medicine, Taipei Veterans General Hospital, Taipei 11127, Taiwan; 6 Faculty of Medicine, National Yang-Ming University School of Medicine, Taipei 11221, Taiwan; 7 Division of Cardiovascular Medicine, Falk Cardiovascular Research Building, Stanford University School of Medicine, Stanford, CA 94305, USA; 8 Institute of Biomedical Sciences, Academia Sinica, Taipei 11529, Taiwan; 9 Graduate Institute of Medical Genomics and Proteomics, National Taiwan University College of Medicine, Taipei 10055 Taiwan; 10 Institute of Epidemiology and Preventive Medicine, College of Public Health, National Taiwan University, Taipei 10055, Taiwan

## Abstract

Proprotein convertase subtilisin/kexin type 2 (*PCSK2*) is a prohormone processing enzyme involved in insulin and glucagon biosynthesis. We previously found the genetic polymorphism of *PCSK2* on chromosome 20 was responsible for the linkage peak of several glucose homeostasis parameters. The aim of this study is to investigate the association between genetic variants of *PCSK2* and glucose homeostasis parameters and incident diabetes. Total 1142 Chinese participants were recruited from the Stanford Asia-Pacific Program for Hypertension and Insulin Resistance (SAPPHIRe) family study, and 759 participants were followed up for 5 years. Ten SNPs of the *PCSK2* gene were genotyped. Variants of rs6044695 and rs2284912 were associated with fasting plasma glucose, and variants of rs2269023 were associated with fasting plasma glucose and 1-hour plasma glucose during OGTT. Haplotypes of rs4814605/rs1078199 were associated with fasting plasma insulin levels and HOMA-IR. Haplotypes of rs890609/rs2269023 were also associated with fasting plasma glucose, fasting insulin and HOMA-IR. In the longitudinal study, we found individuals carrying TA/AA genotypes of rs6044695 or TC/CC genotypes of rs2284912 had lower incidence of diabetes during the 5-year follow-up. Our results indicated that *PCSK2* gene polymorphisms are associated with pleiotropic effects on various traits of glucose homeostasis and incident diabetes.

Development of type 2 diabetes (T2DM) is characterized by tissue resistance to insulin action and failure of pancreatic beta cells to secrete insulin to maintain glucose homeostasis^1^. Both genetic and environmental factors influence susceptibility to T2DM^2^. However, identification of the genes responsible for T2DM is complicated by the high degree of genetic heterogeneity, the involvement of multiple genes, and a small to moderate risk conferred by each of the genes. Searching for quantitative trait loci (QTLs) that explain the variation in the “intermediate” phenotypes of T2DM has therefore been considered a plausible way to tease out the genetic factors involved in biological pathways that lead to development of diabetes^3^. So far, a number of genome-wide linkage scans have been carried out to identify the QTLs for the related intermediate phenotypes of T2DM in Pima Indian^4^, Mexican Americans^5^,^6^, Han Chinese^7^,^8^, Japanese Americans^9^, Caucasians^10^, European Americans and African Americans^11^, and multiethnic population^12^. A QTL for the age of onset of T2DM was identified in a set of French families^13^. However, the putative genetic variants for most of the reported QTLs are largely unknown, including ours^7^.

Calpain 10 (*CAPN10*)^14^, ectonucleotide pyrophosphatase phosphodiesterase 1 (*ENPP1*)^15^, hepatocyte nuclear factor 4α (*HNF4A*)^16^,^17^, adiponectin (*ADIPOQ*)^18^, and transcription factor 7-like 2 (*TCF7L2*)^19^ were previously identified in T2DM-linked chromosomal regions. Using a genome-wide association approach, more than 60 genetic loci to date have been identified for T2DM^20^,^21^. However, the overall fraction of these newly identified T2DM genes remains small, accounting for 5–10% of the heritability of T2DM^22^.

We previously identified a QTL located at 37 cM on chromosome 20 for the fasting insulin and insulin resistance index by homeostasis model assessment (HOMA-IR) in 1,365 non-diabetic Chinese subjects from 411 nuclear families^7^. Following subsequent fine mapping, we found the genetic polymorphism of proprotein convertase subtilisin/kexin type 2 (*PCSK2*) was responsible for the linkage peak of fasting insulin, HOMA-IR, and glucose levels at 1 hr after 75 gm oral glucose loading (data not shown). Therefore, in this study we focused on *PCSK2*.

The protein coded by this gene, *PCSK2*, is a prohormone processing enzyme that plays a key role in regulating insulin and glucagon biosynthesis. *PCSK2* is expressed in the brain and the pancreatic islets. Variants of the *PCSK1* and *PCSK2* genes previously have been linked to T2DM and obesity^23^,^24^,^25^,^26^,^27^. The aim of the current study is to investigate the association between genetic variants of *PCSK2* and fasting insulin and glucose concentration, the homeostasis model assessment of beta cell function (HOMA-beta) and HOMA-IR, various parameters during the 75 g oral glucose tolerance test (OGTT), and clinical progression from normal glucose tolerance to diabetes after a follow-up of 5 years.

## Results

### Characteristics of the SAPPHIRe Chinese participants at baseline and after 5-yr follow- up

A total of 1144 Chinese participants were recruited from the SAPPHIRe study at baseline, and 759 of them received 5-year follow-up examinations. The anthropometric characteristics, plasma glucose and insulin concentration during OGTT, HOMA-IR and HOMA-beta at baseline and after a 5-year follow-up are shown in Table 1.

### Association of PCSK2 genetic variants with various traits of glucose homeostasis

The SNPs and their location in the *PCSK2* gene, their position in the physical map and minor allele frequency are shown in Supplementary Table S1. All the following traits of glucose homeostasis were adjusted for age, gender and body mass index (BMI) and analyzed by family-based association test (FBAT). Genetic variants of rs6044695 and rs2284912 were negatively associated with fasting plasma glucose (FPG) concentration. Genetic variants of rs2269023 were positively associated with FPG and 1-hour plasma glucose concentration (1 h-PG) during OGTT (Table 2). All associations with a q value < 0.05 were considered statistically significant.

### Association of SNP haplotypes of PCSK2 with various traits of glucose homeostasis

**Figure 1:**
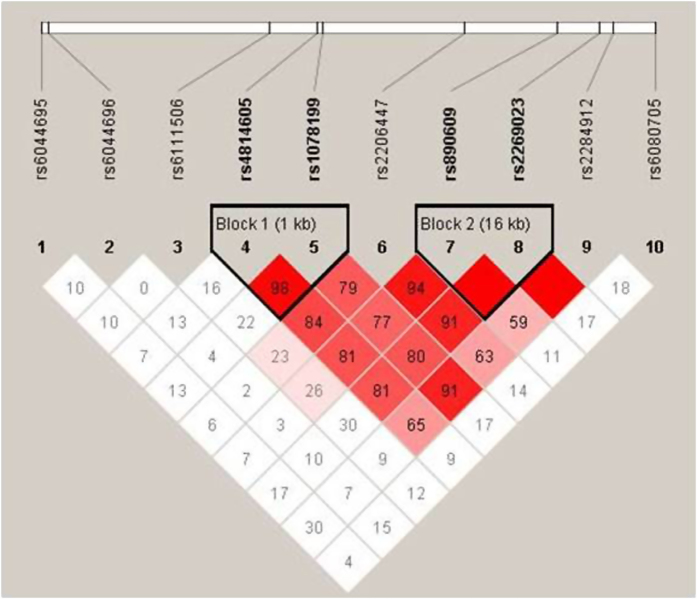
Haploview LD graph of the *PCSK2* gene (10 genotyped SNPs in this study). Pairwise LD coefficients D′ × 100 are shown in each cell (D′ values of 1.0 are not shown). The standard color scheme of Haploview was used for the LD color display (logarithm of likelihood odds ratio [LOD] (a measure of confidence in the value of D′) ≥2 and D′ = 1, shown in bright red; LOD ≥ 2 and D′ < 1 shown in blue; LOD < 2 and D′ = 1 shown in pink; LOD < 2 and D′ < 1 shown in white).

After analysis with Haploview 4.1, the 10 tag SNPs of *PCSK2* were divided into 2 haplotype blocks (block 1: rs4814605/rs1078199, block 2: rs890609/rs2269023, Fig. 1). Haplotypes of rs4814605/rs1078199 (block 1) were associated with fasting plasma insulin concentration (FINS) and HOMA-IR. Haplotypes of rs890609/rs2269023 (block 2) were associated with FPG, FINS and HOMA-IR (Table 3). Both the haplotype-specific P value and global P value were derived from permutation testing 10,000 times. A null hypothesis was rejected if the permuted global *P* value was <0.05.

### Specific SNPs of PCSK2 were associated with progression from normoglycemia to diabetes during a 5-year follow-up

We further used the proportional hazard model to analyze whether the presence or absence of specific SNPs was associated with the progression from normoglycemia to diabetes during a 5-year follow-up. All the p values were adjusted for age, sex, center, drug, environmental factors (including smoking, drinking and sedentary lifestyle), and BMI. The individuals with TA or AA genotypes of rs6044695, or TC or CC genotypes of rs2284912 had a significantly lower incidence of diabetes (Table 4). As shown in Table 5, rs4814597, rs1609659, rs2208203, and rs2021785 were also associated with type 2 diabetes or glucose homeostatic traits according to GWAS database^28^. Since we did not have genotype data of the four SNPs in this study, we reexamined these additional established loci from GWAS in Table 5 by imputing their genotypes using MACH imputation package^29^,^30^ based on 1000 Genomes data. The results are presented in Supplementary Table S2 with a serial number starting with A. None of the imputed SNPs showed evidence of association with incident diabetes. The Haploview linkage disequilibrium (LD) graph of the *PCSK2* gene (10 genotyped SNPs in this study and 4 imputed SNPs: rs4814597, rs1609659, rs2208203, and rs2021785) was shown in Supplementary Fig S1.

## Discussion

In our study, significant associations between some SNPs as well as haplotypes of *PCSK2* and various traits of glucose homeostasis, including FPG, 1 h-PG, FINS and HOMA-IR, were found. Furthermore, individuals with some specific SNPs of *PCSK2* were also associated with progression to diabetes during a 5-year follow-up. In our previous study^31^, we reported the potential pleiotropy of the locus at 37 cM on chromosome 20 on each pair of traits, such as fasting insulin/HOMA-beta and HOMA-IR/HOMA-beta, which supports our present findings that *PCSK2* gene polymorphisms are associated with pleiotropic effects on these metabolic variables.

*PCSK2* is a type II proinsulin-processing enzyme, and it cleaves the proinsulin molecule on the COOH-terminal side of dibasic peptide, Lys64-Arg65, which joins the C-peptide and A-chain domains^32^. Defects affecting the catalytic activity of the prohormone-processing enzymes have been found to be associated with obesity and other metabolic disorders^33^,^34^. The etiology of hyperproinsulinemia is thought to be pancreatic β cell dysfunction, which is manifested in part by inadequate cleavage of proinsulin. Previous studies have shown that increased concentrations of proinsulin are a significant predictor of the development of T2DM in several ethnic groups^35^,^36^,^37^,^38^. Furuta *et al.*^39^ reported that increased levels of proinsulin and split proinsulin were detected in pancreatic islet cells isolated from homozygous *pcsk2* null mice.

There have been several studies reporting that genetic polymorphisms of *PCSK2* were associated with either T2DM or various glucose homeostasis parameters (Table 5). A significant difference in the allele frequency distribution of a simple CA tandem-repeat DNA polymorphism (STRP) in intron 2 of *PCSK2* has been reported in a case-control study of T2DM patients and normal controls in a Japanese population^26^ (Table 5). Jonssan *et al.* recently reported that the C allele of *PCSK2* rs2208203 in intron 2 was associated with reduced insulin secretion measured as the corrected insulin response as well as disposition index^40^. The variant was also associated with lower fasting glucagon levels in non-diabetic individuals with FPG over 5.5 mmol/l^40^ (Table 5). The above microsatellite and rs2208203 in intron 2 were not examined in this study. According to imputation analysis based on 1000 Genomes data, rs2208203 was not associated with incident T2DM.

A more recent genome-wide association study (GWAS) on T2DM in African American families also showed linkage to chromosome 20p in a subset with a later age at diagnosis. The *PCSK2* gene is within the 1-logarithm of odds (LOD) interval of this linkage peak. Association with T2DM was observed among 4 SNPs: rs2021785, rs1609659, rs4814597 and rs2269023^25^ (Table 5). A recent report showed that an association of the risk allele of rs2021785 at *PCSK2* with T2DM also existed in a Han Chinese population^27^ (Table 5). Rs2021785, rs1609659, and rs4814597 were not genotyped in this study. According to imputation analysis based on 1000 Genomes data, the above three imputed SNPs were not associated with incident T2DM. Consistently, in this study, rs2269023 was associated with FPG and 1-hour PG during OGTT in a non-diabetic Han Chinese population (Table 2). Therefore, rs2269023 may play an important role in the regulation of glucose homeostasis in different ethnic groups.

We further searched the open GWAS Central database^28^ for associations between the 10 genetic variations of *PCSK2* investigated in this study and related metabolic phenotypes in Caucasian populations. Significant associations were found between rs2206447 and T2DM (P = 0.008, FUSION Study), and between rs6080705 and HOMA-beta (P = 0.008588), HOMA-IR (P = 0.02582) and fasting insulin (P = 0.01508) (https://www.gwascentral.org/, searched on 6.10.2014) (Table 5). However, these associations could not be replicated in a Han Chinese population in this study. Furthermore, genetic variants of rs6044695 and rs2284912 were associated with both baseline FPG and progression of T2DM during the 5-year follow-up in this study. Therefore, the association at baseline was also replicated in the longitudinal follow-up study. To the best of our knowledge, this is the first study to report that the genetic variants of *PCSK2* were associated with incident T2DM.

This study has several strengths. First, our study used a family-based design, which is a systemic approach to capture all common genetic variations, to control for population stratification. Second, we adopted q-values as our measure of significance in order to reduce false-positive results derived from multiple tests. The q-value is an false-discovery rate (FDR)-based measure of significance used in genome-wide studies. Most importantly, a systematic use of q-values in genome-wide tests of significance will yield a clear balance of false-positive results to true-positive results and provide a standard measure of significance that can be universally interpreted^41^. Third, this study examined SNPs associated with incidence of diabetes rather than prevalence. The limited number of diabetes incidences would be the limitation of this study though.

In conclusion, several genetic variants and haplotypes of *PCSK2* were associated with various traits of glucose homeostasis and progression to diabetes. These findings, together with several earlier observations in different ethnic groups, support an involvement of the *PCSK2* gene in the pathogenesis of T2DM.

## Methods

### Study population of the SAPPHIRe study cohort

The Stanford Asia-Pacific Program for Hypertension and Insulin Resistance (SAPPHIRe) was a collaborative study that was part of the Family Blood Pressure Program of the National Heart, Lung and Blood Institute of the National Institutes of Health meant to investigate the genetic determinants of hypertension and insulin resistance in Chinese and Japanese. The study collected over 1,300 sib pairs that were either concordant or discordant for high blood pressure. Detailed descriptions of the study cohort were published in our previous work^42^,^43^. In brief, subjects were aged between 35 and 60 years and of Chinese or Japanese ancestry. Hypertension was defined as systolic blood pressure >160 mm Hg, diastolic blood pressure >95 mm Hg, or use of 2 medications for high blood pressure (stage II hypertension). Also, the subjects could be taking one medication for high blood pressure with a systolic blood pressure >140 mm Hg or a diastolic blood pressure >90 mm Hg. Low-normal blood pressure was defined as blood pressure in the bottom 30% of the age- and sex-adjusted blood pressure distribution. Individuals with chronic illnesses like diabetes, cancer, or diseases of the heart, liver, or kidney were excluded. In this study, 1142 Chinese participants were recruited from the SAPPHIRe study, and 759 participants received a 5-year follow-up. The institutional review board of each participating site (National Taiwan University Hospital, Taipei Veterans General Hospital, Taichung Veterans General Hospital, and Tri-Serve General Hospital) approved all the experiments in this study. Informed consent was obtained from all subjects.

### Phenotyping

The participants underwent anthropometric measurements at 8 A.M. after an 8–10 h overnight fast. Each subject was subjected to a 75-g OGTT after the anthropometric measurements. Fasting blood samples were collected for the measurement of plasma glucose and insulin. Then, 75 g glucose monohydrate (in 300 ml water) was administered to the subject to drink within 5 minutes. Blood samples were taken for plasma glucose and insulin 1 and 2 hours after glucose loading. The patients were not allowed to eat or drink until the end of the test^7^. Plasma glucose and insulin levels were measured as described previously^7^. HOMA-IR and HOMA-beta derived from the homeostasis model were identical to the previous study^7^.

### Selection of tagSNPs and genotyping

To identify common tagSNPs, we selected tagSNPs from the HapMap CHB (Han Chinese in Beijing) database (phase 1&2, build 35) (http://www.hapmap.org)^44^ using the Tagger program implemented in Haploview version 4 (http://www.broad.mit.edu/mpg/haploview/)^45^. Ten SNPs were selected with minor allele frequencies of more than 10% at *r*^2^ = 0.7, and that captured 80% of alleles of *PCSK2*. SNP genotyping was performed using the GenomeLab SNPstream genotyping platform (Beckman Coulter, Fullerton, CA) and its accompanying SNPstream software suite. ASPEX software was applied to examine Mendelian inconsistencies. When an error was found, the marker data were converted to missing; less than 1% of the marker data were converted to missing in this study. All the methods were carried out in accordance with the approved guidelines. All experimental protocols were approved by committee of National Taiwan University Hospital, Taipei Veterans General Hospital, Taichung Veterans General Hospital, and Tri-Serve General Hospital.

### Statistical analysis

All data were summarized as mean values ± S.D. unless otherwise specified. Pairwise linkage disequilibrium (LD) measures D′ and *r*^2^ were estimated to assess LD between SNPs in the *PCSK2* gene. The structure of the haplotype block was evaluated using the confidence interval method developed by Gabriel *et al.* and implemented in the Haploview program^45^. The association of *PCSK2* SNP and haplotypes with metabolic phenotypes was analyzed using the family-based association test (FBAT)^46^. The trait residuals were obtained based on the generalized linear models adjusted for age, gender, center, drug, environmental factors (i.e., smoking, drinking and sedentary lifestyle), and BMI, then imported into FBAT for association analysis. For each association, we derived a q-value^41^ that was calculated using the statistical package SAS version 9.1. The q-value has been proposed as a FDR-based measure of significance for multiple testing^41^. FDR is the expected proportion of Type I errors among the rejected hypotheses. Q*-value* is defined as an analog of the *p*-value that incorporates FDR-based multiple testing correction^41^. Namely, q-value is the minimum FDR that can be attained to reach significance (i.e., expected proportion of false positives incurred for significance). A p-value of 0.05 implies that 5% of all tests will result in false positives, while an FDR adjusted p-value (or q-value) of 0.05 implies that 5% of significant tests will result in false positives.

We also used the proportional hazard model to analyze whether the presence or absence of specific SNPs was associated with the progression from normoglycemia to diabetes during a 5-year follow-up. A null hypothesis was rejected if the q-value was <0.05. We presented the hazard ratio of the allelic effect from the major allele (A) for each SNP based on Cox regression models. A Cox regression model is a regression-based method for exploring the associations between survival data and explanatory variables. It provides an estimate of the hazard ratio and its confidence interval between two groups. In the present study, the survival data is the person-years for diabetes incidence during the 5-year follow-up period and the explanatory variable of interest is individual SNPs. Proportional hazards regression assumes the hazard ratio is constant over time. Therefore, we conducted Schoenfeld’s residuals test^47^ to check the proportional hazard assumption for each SNP. None of the proportional hazard assumption was rejected suggesting the assumption is legitimate for all the SNPs in the Cox regression analysis (Supplementary Table S3).

We obtained haplotype-specific and whole marker *P*-value by a permutation test. Ten-thousand times were permuted when analyzing family-based association test of *PCSK2* haplotypes with various traits of glucose homeostasis. To calculate permutation-based *P* values, the phenotype labels are randomly shuffled, and all the multiple tests are recalculated emperically on the reshuffled data set, with the smallest *P* value of these multiple tests. The procedure is repeated for 10,000 times to construct an empirical frequency distribution of the smallest *P* values. If the *P* value calculated for the actual data set is smaller than *r* of the 10,000 smallest *P* value from the permuted data sets, then an empirical adjusted P value (P*) is given by P* = (*r* + 1)/(n + 1), where n is the number of replicate samples that have been simulated and *r* is the number of these replicates that produce a test statistic greater than or equal to that calculated for the actual data. A null hypothesis was rejected if the permuted *P* value was <0.05^48^.

## Additional Information

**How to cite this article**: Chang, T.-J. *et al.* Genetic polymorphisms of *PCSK2* are associated with glucose homeostasis and progression to type 2 diabetes in a Chinese population. *Sci. Rep.* **5**, 14380; doi: 10.1038/srep14380 (2015).

## Supplementary Material

Supplementary Information

## Figures and Tables

**Table 1:** Characteristics of the SAPPHIRe Chinese participants.

Variables	Baseline		Follow-up	
	N	Mean ± SD	N	Mean ± SD
Age	1144	48.35 ± 9.09	759	53.18 ± 9.51
Gender (male, %)	537	46.94	348	45.85
BMI (kg/m^2^)	935	24.96 ± 3.43	678	25.46 ± 3.49
FPG (mmol/l)	1136	88.52 ± 10.62	671	95.98 ± 23.09
1 h-PG (mmol/l)	1040	166.45 ± 40.39	629	187.99 ± 49.58
2 h PG (mmol/l)	1023	127.98 ± 32.47	632	148.55 ± 52.03
FINS (pmol/l)	1133	7.37 ± 5.47	674	6.58 ± 5.41
1 h INS (pmol/l)	1039	77.26 ± 56.3	632	84.53 ± 66.83
2 h INS (pmol/l)	1042	61.5 ± 55.58	633	72.39 ± 66.75
HOMA-IR	1132	1.65 ± 1.35	669	1.63 ± 1.56
HOMA-beta	1112	117.76 ± 119.59	663	90.03 ± 144.45
AUCg (mmol*hr/l)	1014	296.4 ± 56.83	629	333.48 ± 82.34
AUCi (pmol*hr/l)	1031	111.88 ± 78.97	NA	NA

BMI: body mass index, AUC_g_: area under curve of plasma glucose, FPG: fasting plasma glucose concentration, 1 h-PG: 1 hr plasma glucose concentration during OGTT, 2 h-PG: 2 hr plasma glucose concentration during OGTT, FINS: fasting plasma insulin concentration, 1 h-INS: 1 hr plasma insulin concentration during OGTT, 2 h-INS: 2 hr plasma insulin concentration during OGTT, AUC_g_: area under curve of plasma glucose, AUC_i_: Area under curve of plasma insulin.

**Table 2:** Family-based association test of *PCSK2* SNPs with glucose homeostasis in SAPPHIRe Chinese.

SNP	FPG			1 h-PG			2 h-PG			FINS			1 h-INS		
	Z	*P*	*Q*	*Z*	*P*	*Q*	*Z*	*P*	*Q*	*Z*	*P*	*Q*	*Z*	*P*	*Q*
rs6044695	−2.71	**0.0068**	**0.023**	−0.36	0.72	0.84	−1.58	0.11	0.77	−1.63	0.10	0.31	−0.18	0.86	0.96
rs6044696	0.62	0.53	0.59	0.20	0.84	0.84	−0.06	0.96	0.96	−1.54	0.12	0.31	−0.80	0.42	0.84
rs6111506	−0.91	0.36	0.59	−1.18	0.24	0.42	−0.41	0.69	0.96	−0.44	0.66	0.87	−0.02	0.98	0.98
rs4814605	−0.55	0.58	0.59	−1.51	0.13	0.42	0.99	0.32	0.77	−1.95	0.051	0.31	−2.04	**0.042**	0.14
rs1078199	−1.10	0.27	0.59	−0.52	0.61	0.84	−1.06	0.29	0.77	−0.25	0.81	0.87	0.19	0.85	0.96
rs2206447	0.80	0.43	0.59	1.14	0.25	0.42	−0.74	0.46	0.77	0.97	0.33	0.59	2.03	**0.042**	0.14
rs890609	−0.54	0.59	0.59	0.27	0.79	0.84	−1.26	0.21	0.77	0.93	0.35	0.59	0.77	0.44	0.84
rs2269023	2.84	**0.0046**	**0.023**	2.95	**0.0032**	**0.032**	0.27	0.79	0.96	1.70	0.088	0.31	2.04	**0.041**	0.14
rs2284912	−2.73	**0.0063**	**0.023**	−1.59	0.11	0.42	−0.14	0.89	0.96	0.19	0.85	0.87	0.67	0.50	0.84
rs6080705	0.54	0.59	0.59	1.20	0.23	0.42	−0.83	0.41	0.77	−0.16	0.87	0.87	0.53	0.60	0.85
SNP	2 h−INS			HOMA−IR			HOMA−beta			AUC_g_			AUC_i_		
	*Z*	*P*	*Q*	*Z*	*P*	*Q*	*Z*	*P*	*Q*	*Z*	*P*	*Q*	*Z*	*P*	*Q*
rs6044695	−0.76	0.45	0.92	−2.02	**0.043**	0.18	−0.18	0.86	0.86	−1.03	0.30	0.55	−0.47	0.64	0.8
rs6044696	−0.91	0.36	0.92	−1.34	0.18	0.45	−1.85	0.064	0.34	0.55	0.58	0.65	−1.16	0.24	0.49
rs6111506	0.21	0.83	0.92	−0.60	0.55	0.79	−1.06	0.29	0.6	−1.23	0.22	0.55	−0.34	0.74	0.82
rs4814605	−0.72	0.47	0.92	−1.93	0.053	0.18	−1.03	0.30	0.6	−0.95	0.34	0.55	−2.27	**0.023**	0.23
rs1078199	0.17	0.86	0.92	−0.47	0.64	0.8	0.86	0.39	0.65	−1.06	0.29	0.55	−0.01	0.99	0.99
rs2206447	0.30	0.76	0.92	1.07	0.28	0.57	0.52	0.60	0.86	0.77	0.44	0.55	1.63	0.10	0.35
rs890609	0.50	0.62	0.92	0.76	0.45	0.75	1.63	0.10	0.35	−0.28	0.78	0.78	0.99	0.32	0.54
rs2269023	0.70	0.48	0.92	2.18	**0.029**	0.18	−0.23	0.82	0.86	2.44	**0.015**	0.15	2.00	**0.045**	0.23
rs2284912	1.69	0.091	0.91	−0.33	0.74	0.82	1.82	0.068	0.34	−1.33	0.18	0.55	1.42	0.16	0.39
rs6080705	−0.10	0.92	0.92	−0.02	0.98	0.98	−0.26	0.80	0.86	0.79	0.43	0.55	0.63	0.53	0.76

All the traits were adjusted for age, gender, center, drug, environmental factors (smoking, drinking and sedentary lifestyle) and BMI.

FPG: Fasting plasma glucose concentration, 1 h-PG: 1 hr plasma glucose concentration during OGTT, 2 h-PG: 2 hr plasma glucose concentration during OGTT, FINS: fasting plasma insulin concentration, 1 h-INS: 1 hr plasma insulin concentration during OGTT, 2 h-INS: 2 hr plasma insulin concentration during OGTT, AUC_g_: area under curve of plasma glucose, AUC_i_: Area under curve of plasma insulin.

**Table 3:**
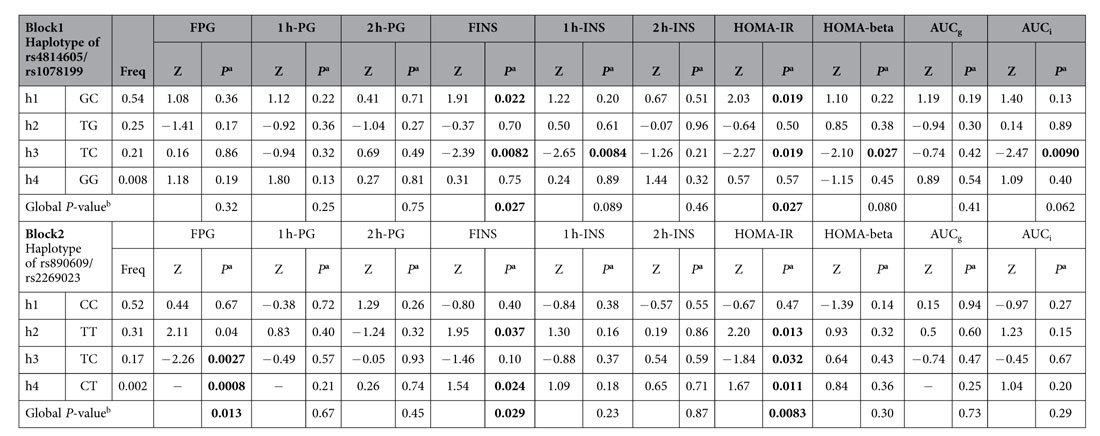
Family-based association test of *PCSK2* haplotypes with various traits of glucose homeostasis in SAPPHIRe Chinese population according to additive model.

All the traits were adjusted for age, gender, and BMI.

^a^Haplotype-specific P-value permutation test 10,000 times.

^b^Whole marker permutation test (chisq sum) 10,000 times; FPG: Fasting plasma glucose concentration; 1 h-PG: 1 hr plasma glucose concentration during OGTT; 2 h-PG: 2 hr plasma glucose concentration during OGTT; FINS: fasting plasma insulin concentration; 1 h-INS: 1 hr plasma insulin concentration during OGTT; 2 h-INS: 2 hr plasma insulin concentration during OGTT; AUCg: area under curve of plasma glucose; AUCi: area under curve of plasma insulin; “–” Z-values unavailable due to sparseness.

**Table 4:** Incidence of progression to diabetes in normoglycemic participants at baseline according to *PCSK2* SNPs using the proportional hazard model.

No.	SNP Name	Major/Minor allele	Incident cases/100 person-year (n = No. of those progressed to diabetes)						Hazard ratio for Aa or aa vs. AA (95% C.I.)*	P** (q)
			AA*		Aa*		aa*			
1	rs6044695	T/A	2.82	(20)	1.33	(11)	0.39	(1)	0.28 (0.12–0.63)	**0.0020 (0.02)**
2	rs6044696	T/C	2	(25)	1.32	(7)	0	(0)	0.32 (0.12–0.83)	**0.019** (0.092)
3	rs6111506	T/A	1.9	(8)	1.95	(18)	1.34	(6)	0.36 (0.15–0.9)	**0.028** (0.099)
4	rs4814605	G/T	1.35	(7)	2.04	(19)	1.75	(6)	0.86 (0.33–2.27)	0.76 (0.98)
5	rs1078199	C/G	1.59	(15)	2.21	(17)	0	(0)	1.05(0.47–2.35)	0.90 (0.98)
6	rs2206447	C/T	2.32	(11)	1.89	(17)	0.95	(4)	0.64 (0.24–1.73)	0.38 (0.98)
7	rs890609	C/T	2.28	(11)	1.89	(17)	0.98	(4)	0.7 (0.25–1.99)	0.51 (0.98)
8	rs2269023	C/T	1.72	(15)	2.07	(15)	1.05	(2)	1.02 (0.34–3.06)	0.98 (0.98)
9	rs2284912	T/C	2.4	(27)	0.65	(4)	2.06	(1)	0.37 (0.16–0.86)	**0.019** (0.092)
10	rs6080705	C/A	1.84	(12)	1.78	(15)	1.7	(5)	1.16 (0.47–2.89)	0.74 (0.98)

^*^AA: homozygote of major allele, Aa: heterozygote of major allele, aa: homozygote of minor allele.

^**^All the P values were adjusted for age, gender, center, drug, environmental factors (smoking, drinking and sedentary lifestyle) and BMI.

**Table 5:** List of *PCSK2* SNPs associated with type 2 diabetes or glucose homeostatic parameters according to published data or GWAS database.

SNP name [Ref. No]	Chromosome Position	Genetic variants	Phenotype	*P*value	Ethnicity
CA repeat in intron 2 polymorphism [ref. 28]	Chromosome 20	(CA)_15–21_	type 2 diabetes	0.0068 [(CA)_21_]	Japanese
rs2208203 [ref. 45]	17,272,003	C/T	reduced insulin secretion and lower fasting glucagon levels in non-diabetic individuals	1.3 × 10^−6^ (corrected insulin response during OGTT) 1.6 × 10^−7^ (disposition index) 0.0048 (fasting glucagon levels)	Finland and Sweden
rs2021785 [ref. 27]	17,370,063	C/T	Type 2 diabetes	0.00014	African Americans
rs1609659 [ref. 27]	17,170,735	A/G		0.028	
rs4814597 [ref. 27]	17,159,633	G/A		0.039	
rs2269023 [ref. 27]	17,381,079	T/C		0.043	
rs2021785 [ref. 29]	17,370,063	C/T	Type 2 diabetes	0.0335	Han Chinese
rs2206447 [ref. 46]	17,330,737	C/T	Type 2 diabetes	0.007631	Caucasian
rs6080705 [ref. 46]	17,401,598	C/A	HOMA-beta	0.008588	
			HOMA-IR	0.02582	
			FINS	0.01508	
