# Supplementary material for: Genetic polymorphisms of PCSK2 are associated with glucose homeostasis and progression to type 2 diabetes in a Chinese population
Source: Sci Rep. 2015 Nov 26;5:14380. doi: 10.1038/srep14380 (PMC4660384; doi:10.1038/srep14380)
Supplement: Supplementary Information [file srep14380-s1.pdf]

Genetic polymorphisms of *PCSK2* are associated with glucose homeostasis and progression to type 2 diabetes in a Chinese population

Tien-Jyun Chang[1,]*, Yen-Feng Chiu[2,]*, Wayne H-H. Sheu[3], Kuang-Chung Shih[4], Chii-Min Hwu[5,6], Thomas Quertermous[7], Yuh-Shan Jou[8], Shan-Shan Kuo[1], Yi-Cheng Chang[1,9], Lee-Ming Chuang[1,10]

Supplementary Fig S1

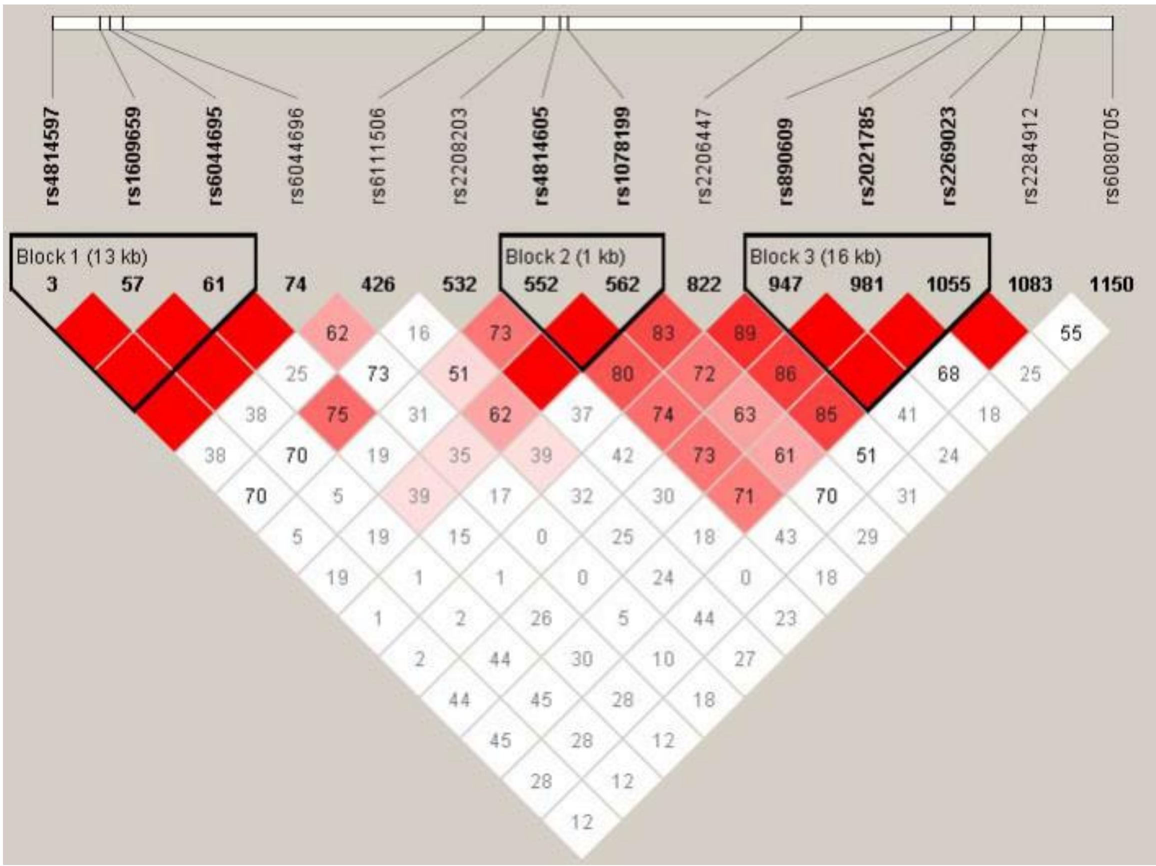

Legend: Haploview LD graph of the *PCSK2* gene (10 genotyped SNPs in this study and 4 imputed SNPs:rs4814597, rs1609659, rs2208203, rs2021785) Pairwise LD

coefficients D'×100 are shown in each cell (D' values of 1.0 are not shown). The standard color scheme of Haploview was used for the LD color display (logarithm of likelihood odds ratio [LOD] (a measure of confidence in the value of D’) ≥2 and D' =1, shown in bright red; LOD ≥2 and D' <1 shown in blue; LOD<2 and D' =1 shown in pink; LOD <2 and D' <1 shown in white).

Supplementary Table S1. SNPs and their location in the *PCSK2* gene, and their position in chromosome 20 according to HapMap Chinese Beijing data base (phase 1&2, build 35)

	SNP Name	Position in chromosome 20	Location in *PCSK2* gene	Major/minor Allele	MAF	HW *P* value
1	rs6044695	17,172,938	intron 1	T/A	0.4	0.63
2	rs6044696	17,175,669	intron 1	T/C	0.24	0.23
3	rs6111506	17,258,016	intron 2	T/A	0.49	0.91
4	rs4814605	17,275,709	intron 2	G/T	0.46	0.48
5	rs1078199	17,277,619	intron_2	C/G	0.26	0.73
6	rs2206447	17,330,737	intron 5	C/T	0.49	0.49
7	rs890609	17,365,013	intron 7	C/T	0.48	0.071
8	rs2269023	17,381,079	intron 8	C/T	0.3	0.48
9	rs2284912	17,386,213	intron 10	T/C	0.2	0.25
10	rs6080705	17,401,598	intron 11	C/A	0.42	0.90

MAF, minor allele frequency; HW P value, *P* value for Hardy–Weinberg equilibrium test.

Supplementary Table S2. Incidence of progression to diabetes in normoglycemic participants at baseline according to imputed 4 SNPs of *PCSK2* gene using the proportional hazard model.

No.	SNP Name	Major/ Minor allele	Incident cases/ 100 person-year (n=No. of those progressed to diabetes)						Hazard ratio for Aa or aa vs. AA (95% C.I.)*	P** (q)
			AA*		Aa*		aa*			
**A1**	**rs4814597**	G/A	2.02	(27)	1.11	(5)	0	(0)	0.83 (0.28-2.47)	0.74 (0.98)
**A2**	**rs1609659**	A/G	1.95	(26)	1.32	(6)	0	(0)	1.12 (0.41-3.06)	0.83 (0.98)
**A3**	**rs2208203**	T/C	1.62	(19)	2.17	(13)	0	(0)	1.23 (0.5-3.01)	0.66 (0.98)
**A4**	**rs2021785**	G/A	1.75	(13)	2.07	(17)	0.88	(2)	0.95 (0.31-2.98)	0.94 (0.98)

*AA: homozygote of major allele, Aa: heterozygote of major allele, aa: homozygote of minor allele

** All the P values were adjusted for age, gender, center, drug, environmental factors (smoking, drinking and sedentary lifestyle) and BMI.

Supplementary Table S3. Checking the proportional hazard assumption for each SNP of *PCSK2* gene by conducting Schoenfeld's residuals test

No.	SNP Name	Major/ Minor allele	P-value for checking proportional hazards assumption
**A1**	**rs4814597**	G/A	0.76
**A2**	**rs1609659**	A/G	0.82
**1**	**rs6044695**	T/A	0.84
**2**	**rs6044696**	T/C	0.47
**3**	**rs6111506**	T/A	0.60
**A3**	**rs2208203**	T/C	0.76
**4**	**rs4814605**	G/T	0.87
**5**	**rs1078199**	C/G	0.86
**6**	**rs2206447**	C/T	0.54
**7**	**rs890609**	C/T	0.34
**A4**	**rs2021785**	G/A	0.74
**8**	**rs2269023**	C/T	0.84
**9**	**rs2284912**	T/C	0.46
**10**	**rs6080705**	C/A	0.83

*AA: homozygote of major allele, Aa: heterozygote of major allele, aa: homozygote of minor allele

** All the P values were adjusted for age, gender, center, drug, environmental factors (smoking, drinking and sedentary lifestyle) and BMI.

The serial number starting with A indicated the imputed SNPs
